# Perineural Invasion Worsens Long-Term Outcomes of Pancreatic Neuroendocrine Tumors Following Surgical Resection

**DOI:** 10.1245/s10434-025-18561-6

**Published:** 2025-10-16

**Authors:** Hui Xu, Jing-Jing Hou, Jun-Xi Xiang, Alexandra G. Lopez-Aguiar, George Poultsides, Flavio Rocha, Sharon Weber, Ryan Fields, Kamran Idrees, Cliff Cho, Shishir K. Maithel, Yi Lv, Xu-Feng Zhang, Timothy M. Pawlik

**Affiliations:** 1 Department of Hepatobiliary Surgery and Institute of Advanced Surgical Technology and Engineering, The First Affiliated Hospital of Xi’an Jiaotong University, Xi’an, China; 2 Division of Surgical Oncology, Department of Surgery, Winship Cancer Institute, Emory University, Atlanta, Georgia; 3 Department of Surgery, Stanford University, Palo Alto, CA USA; 4 Department of Surgery, Virginia Mason Medical Center, Seattle, WA USA; 5 Department of Surgery, School of Medicine and Public Health, University of Wisconsin, Madison, WI USA; 6 Department of Surgery, Washington University School of Medicine, St. Louis, WI USA; 7 Division of Surgical Oncology, Department of Surgery, Vanderbilt University, Nashville, TN USA; 8 Division of Hepatopancreatobiliary and Advanced Gastrointestinal Surgery, Department of Surgery, University of Michigan, Ann Arbor, MI USA; 9 Division of Surgical Oncology, The Ohio State University Wexner Medical Center and James Comprehensive Cancer Center, Columbus, OH USA

## Abstract

**Background:**

To define the impact of perineural invasion (PNI) on long-term survival of patients following curative-intent resection of pancreatic neuroendocrine tumors (pNETs).

**Patients and Methods:**

Patients with pNETs who underwent curative-intent resection (R0/R1) between 2000 and 2020 were identified from a multi-institutional database. The impacts of PNI on overall survival (OS) and disease-free survival (DFS) were analyzed.

**Results:**

Among 700 patients, 171 (*n* = 24.4%) had a pNET with PNI. The presence of PNI was associated with higher tumor grade (G3, 8.2% vs. 2.5%, *p* < 0.001), more advanced AJCC T disease (T3–T4, 58.5% vs. 15.9%, *p* < 0.001), and a higher incidence of nodal metastasis (52.6% vs. 21.2%, *p* < 0.001) versus patients with no PNI. Patients with PNI had a worse OS (median, with PNI 115.9 months vs. no PNI not reached, *p* < 0.001) and DFS (median, with PNI 51.9 vs. no PNI 115.4 months, *p* < 0.001) versus patients with no PNI. On multivariable analysis PNI was an independent risk factor associated with worse OS (HR = 2.624, 95%CI 1.475–4.668, *p* = 0.001), as well as DFS (HR = 1.972, 95%CI 1.396–2.786, *p* < 0.001). Among 256 patients with very early staged tumors (G1N0) who underwent an R0 resection, PNI remained a strong independent factor associated with worse long-term survivals (OS, HR = 3.892, 95%CI 1.196–12.662, *p* = 0.024; DFS, HR = 2.530, 95%CI 1.010–6.339, *p* = 0.048).

**Conclusions:**

PNI was an independent adverse prognostic factor among patients undergoing curative-intent resection of pNETs, even among individuals with early-stage disease. The presence of PNI should be routinely assessed and considered in the prognostic stratification of patients following resection of pNETs.

**Supplementary Information:**

The online version contains supplementary material available at 10.1245/s10434-025-18561-6.

Pancreatic neuroendocrine tumors (pNETs), originating from pancreatic islet cells of Langerhans, represent a rare malignancy accounting for 2–10% of all pancreatic neoplasms.^1^ Although pNETs typically exhibit indolent behavior with slow progression, a distinct subset displays clinically aggressive phenotypes manifesting as locoregional and distant metastases or disease recurrence after curative-intent resection.^2^ Patients with worse long-term outcomes usually have pNETs characterized by a high tumor burden, poor tumor grade, as well as lymph node and/or liver metastases.^3–6^ However, the accuracy and overall discriminatory ability of these individual factors remain limited, as metastatic dissemination and disease recurrence occur even among patients with early stage disease such as G1/G2 tumor, no microvascular invasion, or no lymph node metastasis.^7,8^ Other factors therefore need to be included in the prognostic stratification and inform adjuvant therapeutic decision-making of patients with pNETs.

Perineural invasion (PNI), defined as tumor cell infiltration into neural structures or perineural spaces,^9^ was historically underestimated in oncobiology as a passive epiphenomenon.^10^ Emerging evidence now implicates PNI as an active driver of tumor progression in pancreatic ductal adenocarcinoma, intrahepatic cholangiocarcinoma, colorectal carcinoma, and prostate cancer with independent prognostic significance.^11–14^ Notably, pNETs exhibit marked neurotropic behavior, wherein neural and perineural invasion may establish a distinct metastatic pathway analogous to lymphatic dissemination,^15^ which potentially underlies postoperative recurrence and lymph node metastasis. Crosstalk between neurons and tumor cells or other stromal cells within the microenvironment has also been reported to promote tumor growth and progression.^16^ However, the prognostic significance of PNI in pNETs remains poorly defined with conflicting evidence regarding its clinical relevance.^17,18^ As such, the objective of the current study was to delineate the impact of PNI on overall survival (OS) and disease-free survival (DFS) among patients following curative-intent resection of pNETs.

## Patients and Methods

### Patient Cohort

Patients with pNETs who underwent curative-intent resection (R0/R1) between 2000 and 2020 were identified from eight institutions (The Ohio State University Wexner Medical Center and James Comprehensive Cancer Center, Columbus, OH; Winship Cancer Institute, Emory University, Atlanta, GA; Stanford University, Palo Alto, CA; Virginia Mason Medical Center, Seattle, WA; University of Wisconsin, School of Medicine and Public Health, Madison, WI; Washington University, School of Medicine, St. Louis, MO; Vanderbilt University, Nashville, TN; University of Michigan, Ann Arbor, MI). The Institutional Review Board of all participating institutions approved the study. A waiver of informed consent was obtained, since the data were analyzed from the electronic medical record and reported without personal identifiers.

**Fig. 1:**
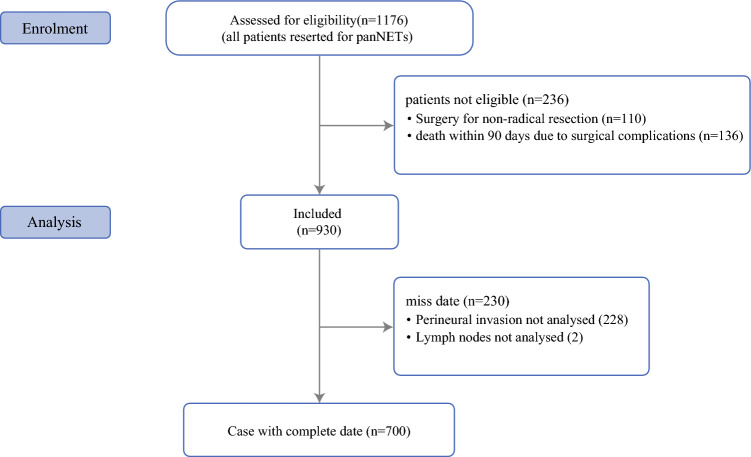
Overview of study population

All patients were diagnosed with pNETs upon postoperative histological examination. Demographics and clinicopathologic information were collected on the basis of a standardized database across each institution. Patients with no detailed information on R status, N status, or PNI, and individuals who died within 90 days following surgery were excluded (Fig. 1).

### Follow-up and Survival

Patients were regularly followed per a standardized protocol that included surveillance every 3–4 months within the first 3 years after resection and then once every 6 months until the fifth year, after which screening occurred annually. Serum tumor markers were monitored along with routine imaging studies. Recurrence was determined by imaging studies and/or biopsy. Overall survival (OS) was defined as time from surgery to death from any cause or last follow-up, and disease-free survival (DFS) was measured from the date of surgery to recurrence (pathologically/radiologically confirmed), death, or last follow-up.

### Statistical Analysis

Baseline variables were summarized descriptively: categorical data as frequencies (%) and continuous variables as median values (interquartile range, IQR). Group comparisons used *χ*^2^/Fisher’s exact tests for categorical variables and *t*-tests/Mann–Whitney *U* tests for continuous measures. OS and DFS were evaluated using Kaplan–Meier curves and compared using log-rank tests. The univariate and multivariable analysis were performed with Cox proportional hazards models and reported as hazard ratios (HR) with 95% confidence intervals (CI). Figures were generated using GraphPad Prism/R software/Bio render. SPSS version 27.0 (SPSS Inc., Chicago, IL, USA) was employed for analyses, with two-tailed *p* < 0.05 denoting significance.

## Results

### Baseline and Clinicopathological Characteristics

Among the 1176 patients who underwent curative-intent resection (R0/R1) of pNETs, 700 patients were included in the analytic cohort (Fig. 1). Median age was 58.4 (48.0–66.5) years, and 367 (52.4%) individuals were male (Table 1). The majority of patients had nonfunctional pNETs (*n* = 599, 85.6%). Most tumors were located in the pancreatic body/tail (*n* = 407, 58.1%) followed by pancreatic head/uncinate (*n* = 256, 36.6%). Moreover, most patients had G1/G2 tumors (*n* = 560, 80.0%); 59.6% (*n* = 417) of patients had a tumor size ≥ 2 cm. On pathological examination, 202 (28.9%) patients had nodal metastasis, whereas a subset of patients (*n* = 37, 5.3%) developed liver metastasis and underwent metastatic tumor resection/ablation simultaneously. Most patients (*n* = 579, 82.7%) achieved R0 resection of the primary pNETs.

**Table 1:** Baseline characteristics

	Total (*n* = 700)	With PNI (*n* = 171)	Without PNI (*n* = 529)	*p* value
Age (years)	58.4 (48.5, 67.0)	59.4 (47.0, 66.8)	58.0 (50.0, 67.0)	0.990
*Gender*				0.100
Male	367 (52.4%)	99 (57.9%)	268 (50.7%)	
Famale	333 (47.6%)	72 (42.1%)	261 (49.3%)	
*ASA class*				0.063
1–2	268 (38.3%)	75 (45.5%)	193 (36.5%)	
3–4	414 (59.1%)	90 (54.5%)	324 (61.2%)	
*BMI (kg/m*^*2*^*)*				0.027
< 25.0	187 (26.7%)	50 (29.2%)	137 (25.9%)	
≥ 25.0	462 (66.0%)	103 (60.2%)	359 (67.9%)	
*Functional tumor*				0.001
No	599 (85.6%)	158 (92.4%)	441 (83.4%)	
Yes	93 (13.3%)	10 (5.8%)	83 (15.7%)	
*Location of tumor*				<0.001
Head	226 (32.3%)	83 (48.5%)	143 (27.0%)	
Uncinate	30 (4.3%)	8 (4.7%)	22 (4.2%)	
Neck	36 (5.1%)	8 (4.7%)	28 (5.3%)	
Body	138 (19.7%)	28 (16.4%)	110 (20.8%)	
Tail	269 (38.4%)	44 (25.7%)	225 (42.5%)	
*Tumor differentiation*				<0.001
Well	577 (82.4%)	117 (68.4%)	460 (87.0%)	
Moderately	58 (8.3%)	22 (12.9%)	36 (6.8%)	
Poorly	14 (2.0%)	10 (5.8%)	4 (0.8%)	
*Tumor grade*				<0.001
G1	364 (52.0%)	59 (34.5%)	305 (57.7%)	
G2	196 (28.0%)	62 (36.3%)	134 (25.3%)	
G3	27 (3.9%)	14 (8.2%)	13 (2.5%)	
*Largest tumor size*				<0.001
< 2 cm	281 (40.1%)	38 (22.2%)	243 (45.9%)	
≥ 2 cm	417 (59.6%)	133 (77.8%)	284 (53.7%)	
*AJCC T category*				<0.001
T1–T2	515 (73.6%)	71 (41.5%)	444 (83.9%)	
T3–T4	184 (26.3%)	100 (58.5%)	84 (15.9%)	
*AJCC N category*				<0.001
N0	498 (71.1%)	81 (47.4%)	417 (78.8%)	
N1	202 (28.9%)	90 (52.6%)	112 (21.2%)	
*AJCC M category*				0.17
M0	657 (93.9%)	156 (91.2%)	501 (94.7%)	
M1	37 (5.3%)	13 (7.6%)	24 (4.5%)	
*Resection margin*				<0.001
R0	579 (82.7%)	121 (70.8%)	458 (86.6%)	
R1	121 (17.3%)	50 (29.2%)	71 (13.4%)	
*Lymphovascular invasion*				<0.001
No	448 (64.0%)	47 (27.5%)	401 (75.8%)	
Yes	236 (33.7%)	115 (67.3%)	121 (22.9%)	

**Fig. 2:**
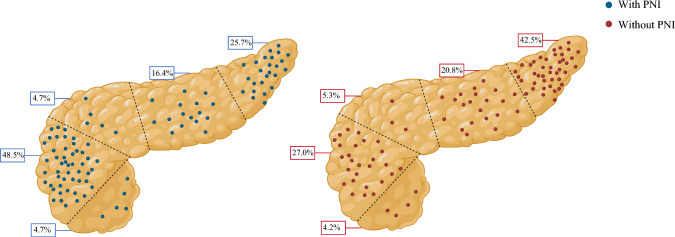
Proportion of patients with perineural invasion at different locations of the pancreas

Among 700 patients, 171 (*n* = 24.4%) had pNETs with PNI (Table 1). The presence of PNI was associated with a higher proportion of nonfunctional tumors (92.4% vs. 83.4%, *p* = 0.001), poor tumor differentiation (5.8% vs. 0.8%, *p* < 0.001), higher tumor grade (G3, 8.2% vs. 2.5%, *p* < 0.001), larger tumor size (≥ 2 cm, 77.8% vs. 53.7%, *p* < 0.001), as well as a higher incidence of lympho-vascular invasion (67.3% vs. 22.9%, *p* < 0.001), more advanced AJCC T disease (T3–T4, 58.5% vs. 15.9%, *p* < 0.001), and a higher incidence of nodal metastasis (52.6% vs. 21.2%, *p* < 0.001) versus patients with no PNI. Of note, 91 (53.2%) patients with PNI had a tumor located in the pancreatic head/uncinate, whereas most patients (*n* = 335, 63.3%) with tumors and no PNI had the tumor located in the body/tail (*p* < 0.001) (Fig. 2).

### PNI and Long-Term Survival

**Fig. 3:**
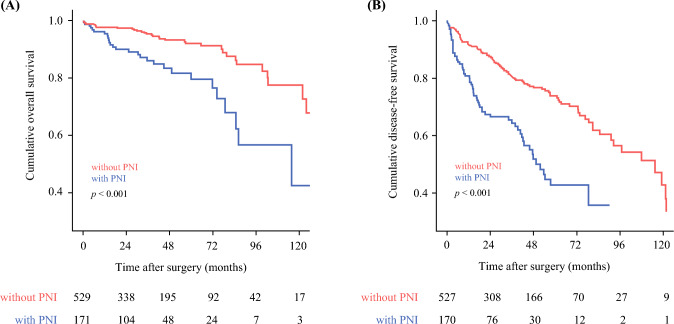
Overall and disease-free survival of patients with versus without perineural invasion in the overall cohort

Overall, 3 and 5 year OS were 94.7% and 93.2%, respectively; 3 and 5 year DFS were 82.1% and 77.9%, respectively. Patients with pNETs characterized by PNI had a worse OS (median, PNI 115.9 months vs. no PNI not attained, *p* < 0.001) and DFS (median, PNI 51.9 vs. no PNI 115.4 months, *p* < 0.001) (Fig. 3a and b). Among patients with an R0 resection, PNI remained associated with a worse long-term survival (Supplementary Fig. 1a and b). In addition, on multivariable analysis PNI was an independent risk factor associated with worse OS (HR = 2.624, 95%CI 1.475–4.668, *p* = 0.001), as well as DFS (HR = 1.972, 95%CI 1.396–2.786, *p* = <0.001)(Table 2). Among the 178 (25.4%) patients who had recurrent disease, most patients developed recurrence in the liver (*n* = 93, 52.2%). In addition, functional pNETs were excluded, PNI remained associated with worse OS (HR = 2.846, 95%CI 1,660–4.882, *p* < 0.001) and DFS (HR = 2.307, 95%CI 1.542–3.453, *p* < 0.001) among patients with nonfunctional pNETs (Supplementary Fig. 2a and b).

**Table 2:** Multivariable regression analysis of risk factors for overall and disease-free survival in the entire cohort

	Univariable analysis		Multivariable analysis	
	Hazard ratio	*p* value	Hazard ratio	*p* value
Overall survival				
*Tumor differentiation*				
Well	Ref		Ref	
Moderately	1.322 (0.524–3.336)	0.555	1.020 (0.401–2.598)	0.966
Poorly	6.224 (2.638–14.688)	<0.001	3.277 (1.342–8.001)	0.009
*Functional tumor*				
No	Ref			
Yes	1.710 (0.732–3.999)	0.215		
*Largest tumor size*				
< 2 cm	Ref		Ref	
≥ 2 cm	2.153 (1.209–3.833)	0.009	1.752 (0.866–3.546)	0.119
*Resection margin*				
R0	Ref			
R1	1.402 (0.796–2.470)	0.242		
*AJCC N category*				
N0	Ref		Ref	
N1	1.970 (1.213–3.199)	0.006	1.364 (0.782–2.381)	0.274
*Perineural invasion*				
No	Ref		Ref	
Yes	2.844 (1.744–4.638)	<0.001	2.624 (1.475–4.668)	0.001
Disease-free survival				
*Tumor differentiation*				
Well	Ref		Ref	
Moderately	0.963 (0.534–1.738)	0.900	0.711 (0.391–1.293)	0.264
Poorly	3.403 (1.731–6.691)	< 0.001	1.842 (0.925–3.669)	0.082
Functional tumor				
No	Ref			
Yes	1.001 (0639–1.568)	0.996		
Largest tumor size				
< 2 cm	Ref		Ref	
≥ 2 cm	3.251 (2.245–4.708)	< 0.001	2.620 (1.718–3.995)	< 0.001
*Resection margin*				
R0	Ref		Ref	
R1	1.636 (1.160–2.308)	0.005	1.148 (0.790–1.670)	0.469
*AJCC N category*				
N0	Ref		Ref	
N1	3.049 (2.271–4.095)	< 0.001	1.970 (1.409–2.754)	< 0.001
*Perineural invasion*				
No	Ref		Ref	
Yes	2.377 (1.747–3.233)	< 0.001	1.972 (1.396–2.786)	< 0.001

### PNI on Long-Term Survival of Early Staged Patients

The impact of PNI on long-term survival among patients with early stage pNETs was examined. Among 497 patients with no nodal metastasis (N0), PNI was associated with worse outcomes (median OS, PNI 57.6 vs. no PNI 115.4 months, *p* = 0.014; median DFS, median 34.9 vs. 47.5 months, *p* = 0.002)(Supplementary Fig. 3a and b). Similarly, among patients with nodal metastasis (N1), PNI remained associated with inferior survival outcomes, including reduced OS (median, PNI 86.3 vs. no PNI 143.0 months, *p* = 0.004) and DFS (median, PNI 38.0 vs. no PNI 63.6 months, *p* = 0.017).

**Fig. 4:**
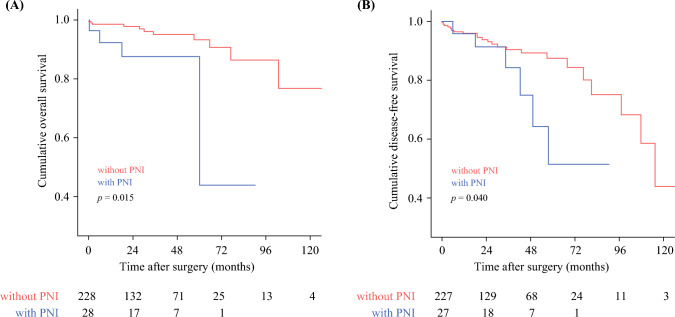
Overall and disease-free survival of patients with no nodal metastasis and G1 disease (N0G1) following an R0 resection stratified by the status of perineural invasion

There were 256 patients with very early staged tumors (G1N0) who underwent an R0 resection. Among these patients PNI remained a strong independent factor associated with worse long-term survival (OS, HR = 3.892, 95%CI 1.196–12.662, *p* = 0.024; DFS, HR = 2.530, 95%CI 1.010–6.339, *p* = 0.048)(Table 3). Of note, among patients who underwent an R0 resection of G1N0 disease, individuals with PNI pNETs had a worse OS (median, PNI 60.1 months versus no PNI not reached, *p* = 0.015) and DFS (PNI not reached versus no PNI 107.9 months, *p* = 0.040)(Fig. 4 a and b).

**Table 3:** Regression analysis of risk factors for overall and disease-free survival in the R0N0G1 cohort

	Univariable analysis	
	Hazard ratio	*p* value
Overall survival		
*Functional tumor*		
No	Ref	
Yes	1.113 (0.313–3.961)	0.869
*Largest tumor size*		
< 2 cm	Ref	
≥ 2 cm	0.870 (0.308–2.458)	0.793
*Perineural invasion*		
No	Ref	
Yes	3.892 (1.196–12.662)	0.024
Disease-free survival		
*Functional tumor*		
No	Ref	
Yes	1.007 (0.383–2.650)	0.988
*Largest tumor size*		
< 2 cm	Ref	
≥ 2 cm	0.822 (0.387–1.747)	0.610
*Perineural invasion*		
No	Ref	
Yes	2.530 (1.010–6.339)	0.048

## Discussion

Tumor recurrence and metastatic dissemination remain important determinants of long-term survival among patients with PanNETs. Prognostic factors including tumor grade, tumor burden, and nodal involvement have been previously validated ^6,19,20^. However, a subset of patients who lack these features and have as localized early-stage disease (AJCC T1–2) still have poor DFS and OS. In turn, current staging paradigms may be inadequate and not account for other biological determinants related to prognosis. As such, the current study was important, as we specifically demonstrated the prognostic role of PNI as an independent adverse factor among patients undergoing curative-intent resection pNETs. In fact, even among patients with traditionally favorable histopathological profiles (e.g., pN0, R0, and G1), PNI was an important determinant of survival—with an over two-fold risk of worse DFS and OS. These findings suggest that PNI was a valuable prognostic biomarker for long-term outcome stratification, particularly among patients with early-stage pNETs who might be misclassified as low-risk on the basis of conventional staging criteria.

Data in the current study was consistent with previous work from Bor-Shiuan Shyr and Wataru Izumo^19,20^ that demonstrated PNI to be a biomarker of aggressive tumor biology. In the current study, patients with pNETs characterized by PNI were more likely to have tumors with poor differentiation and higher tumor grade. Beyond conventional hematogenous and lymphatic routes, PNI may facilitate locoregional infiltration and distant metastasis via perineural spaces.^15,21^ PNI constitutes an active tumor-neural interaction wherein cancer cells exploit nerve tracts as avenues of distant spread. Furthermore, PNI-induced neurogenic inflammation triggers the release of pro-angiogenic factors (e.g., vascular endothelial growth factor, nerve growth factor), simultaneously promoting neoangiogenesis and lymphangiogenesis thereby facilitating metastatic conduits. This mechanism may explain, in part, why pNETs characterized by PNI had a heavier tumor burden; PNI may be a microscopic manifestation of tumors with a propensity for early dissemination, and are more likely to be associated with lymphovascular invasion and lymph node metastasis. Neurons actively interact with cancer cells and stromal cells (including fibroblasts, endothelial cells, and immune cells) in the tumor microenvironment, promoting tumor growth and metastasis^22^. Interestingly, PNI-positive tumors demonstrated a predilection for the pancreatic head, suggesting potential interactions between neural invasion propensity and region-specific microenvironmental factors, such as denser peripancreatic neural plexuses in the pancreatic head. Collectively, PNI could serve as a strong adjuvant biomarker of aggressive disease biology, independent of other tumor or stage-specific factors.

While PNI affected the prognosis of the entire cohort, when stratified by T and N categories, as well as tumor grade, and adjusted for confounding factors, the presence of PNI remained associated with unfavorable OS and DFS even among patients with early-stage disease. Patients with AJCC T1–2, no lymph nodes, and G1 grade tumors are generally considered to have favorable histopathological features.^3,18,23^ Data in the current study demonstrated, however, that 13.8% of patients with stage T1–2 had PNI; the incidence of PNI was 16.3% and 16.2%, respectively, among patients with N0 and G1 disease. In fact, the data demonstrated that PNI not only occurred independently of lymphovascular invasion and tumor cell proliferation but also may serve as an independent metastatic pathway. For example, differences in survival were noted on the basis of stratification relative to PNI among early-stage patients. In addition, PNI remained strongly predictive of OS on multivariable analysis after controlling for other risk factors.

Although recent guidelines have proposed observation as an optional treatment strategy for small, low-grade pNETs^24,25^, the presence of PNI should be taken into account. In particular, survival outcomes of patients who were previously thought to have relatively favorable pathological characteristics may have a more guarded prognosis in the setting of PNI. To this point, the presence of PNI was associated with OS and DFS even among patients who had undergone an R0 resection. This finding highlights the potential role of PNI as an important biomarker in early-stage patients without other high-risk features (such as larger tumor volume or lymph node metastasis). PNI may reflect residual micrometastases or immune dysregulation, making patients prone to recurrence. Therefore, incorporating PNI into the risk assessment system for early pNETs patients can help identify “seemingly” early but actually high-risk patient groups. For these early-stage patients with positive PNI, more frequent follow-up monitoring (such as more frequent imaging examinations) or even the potential value of adjuvant therapy may be necessary to detect or prevent the occurrence of metastatic diseases earlier with the goal of improving long-term outcomes.

The current study had several limitations. As a retrospective study, there may have been selection and information bias. The diagnosis of PNI was determined on the basis of the pathologist’s judgment and lacked quantitative criteria (such as neural invasion density, the diameter of affected nerve bundles, or spatial distribution patterns). The reading pathologists were, however, at high volume pancreatic centers with extensive experience. In the future, development of artificial intelligence-based tools to preoperatively integrate imaging data and construct predictive models for PNI holds clinical promise to assess the risk of tumor metastasis, thereby providing a more comprehensive basis for decision-making.^26–29^ In addition, while PNI-positive tumors had a worse OS and DFS, effective adjuvant therapy for pNETs remains somewhat lacking. In the future, small molecule inhibitors or antibody drugs that specifically block neural invasion, targeting the neuro-tumor interactions, will hopefully emerge as new therapeutic targets for pNETs.

In conclusion, PNI was an independent adverse prognostic factor among patients undergoing curative-intent resection of pNETs, even among individuals with early-stage disease. The presence of PNI should be routinely assessed and considered in the prognostic stratification of patients following resection of pNETs. Further studies are needed to identify the molecular mechanisms of PNI in pNETs with the aim to develop new therapeutic targeted therapy.

## Supplementary Information

Below is the link to the electronic supplementary material.

Supplementary file1 (DOCX 260 kb)
