# Supplementary material for: Perineural Invasion Worsens Long-Term Outcomes of Pancreatic Neuroendocrine Tumors Following Surgical Resection
Source: Ann Surg Oncol. 2025 Oct 16;33(2):1586–94. doi: 10.1245/s10434-025-18561-6 (PMC12765729; doi:10.1245/s10434-025-18561-6)
Supplement: Supplementary file 1 — Supplementary file1 (DOCX 260 kb) [file 10434_2025_18561_MOESM1_ESM.docx]

**Supplementary Figure 1.** Overall and disease-free survival of patients following R0 resection of pancreatic neuroendocrine tumors stratified by perineural invasion status.

**Supplementary Figure 2.** Overall and disease-free survival of patients with non-functional pancreatic neuroendocrine tumors stratified by perineural invasion status.

**Supplementary Figure 3.** Overall and disease-free survival of N0 patients stratified by perineural invasion status.
